# Frequency of HIV and HCV Co-Infections in Chronic HBV Patients Referred to Taleghani Hospital, Tehran, Iran from 2006 to 2010

**DOI:** 10.5812/kowsar.1735143X.740

**Published:** 2011-12-20

**Authors:** Seyed Mohammad Ebrahim Tahaei, Seyed Reza Mohebbi, Pedram Azimzadeh, Mohsen Vahedi, Shohreh Almasi, Sara Romani, Afsaneh Sharifian, Faramarz Derakhshan, Mohammad Reza Zali

**Affiliations:** 1 Research Center for Gastroenterology and Liver Diseases, Shahid Beheshti University of Medical Sciences, Tehran, IR Iran

**Keywords:** Epidemiology Frequency, Human Immunodeficiency Virus, Hepatitis C, Hepatitis B, Co-infection

## Abstract

**Background:**

Co-infection with hepatitis C virus (HCV) and/or human immunodeficiency virus (HIV) in patients with chronic hepatitis B virus (HBV) infection can alter the course of the disease.

**Objectives:**

In this study, we investigated the frequency of HIV and/or HCV co-infection in chronic HBV patients and related risk factors in acquiring the HCV and or HIV co-infectionit.

**Patients and Methods:**

We studied 264 chronic HBV patients who visited the Gastrointestinal and Liver Ward of the Taleghani Hospital, Tehran, Iran between 2006 and 2010. Demographic information and records of possible risky behavior were obtained. Antibodies against HBV, HCV, and HIV, levels of alanine transaminase (ALT) and aspartate transaminase (AST), and conversion from hepatitis B e antigen (HBeAg) to hepatitis B e antibody (HBeAb) were evaluated.

**Results:**

Of 264 patients with chronic HBV in this study, 184 patients (70%) were men and 78 patients (30%) were women. Only 1 patient (0.37%) was positive for anti-HIV antibody, whereas 12 patients (4.54%) were positive for anti-HCV antibody. None of the patients had co-infection with all 3 viruses (HBV, HIV, and HCV).

**Conclusions:**

This study demonstrated that the prevalence of HCV is higher than that of HIV in chronic HBV patients. Since HCV or HIV co-infection affects the therapeutic outcome in chronic HBV patients, testing for HIV and HCV is recommended, especially for patients with a history of risky behavior.

## 1. Background

Human immunodeficiency virus (HIV), hepatitis C virus (HCV), and hepatitis B virus (HBV) are among the most important infectious agents in the world and are considered a noteworthy problem. These viruses have similar epidemiologic properties and transmission routes. About 2 billion people worldwide have been infected with HBV and about 350 million are chronically infected [1]. In 1979, the prevalence of HBV was 2.2-7% in the Iranian population [2], but according to recent reports, the prevalence of HBV in Iran has decreased to less than 2% [3]. HCV has infected 170 million people, approximately 3% of the world population [4]. According to a recent study, 0.093% of the Iranian blood donor population is infected with HCV [5]. This infection progresses into chronic disease and eventually leads to cirrhosis and sometimes hepatocellular carcinoma. HIV is the causative agent of Acquired Immunodeficiency Syndrome AIDS and although there has been significant progress in diagnosis, pathogenesis, and treatment of this disease, a preventive vaccine or absolute cure for this disease remains out of sight. Just in 2007, 2 million people died because of AIDS; in the same year, 2.7 million people were infected by this virus [6]. Of the blood donor population in Iran, 0.003% are infected with this virus [5].

Almost one third of HIV-infected patients in Europe and America are co-infected with HCV, and 10% of HIV-infected patients are co-infected with HBV [7]. In a recent study performed in Iran, researchers found that the seroprevalence in the general population was 0.56% for HBV, 0.13% for HCV, and 0.004% for HIV [8]. With regard to co-infections, most studies in Iran have been performed on inmates or hospitalized drug abusers. These studies showed that HBV-HCV co-infection is frequent, whereas triple co-infection was never observed [9][10][11]. Co-infection of HBV with HCV or HIV could play a critical role in the course of the disease and efficacy efficiency of treatment [7]. Therefore, evaluation of chronic HBV patients for co-infection with HCV or HIV has great importance for physicians in choosing a treatment regimen and in considering disease progression

## 2. Objectives

There is scarce information on the prevalence of co-infection with HCV or HIV in Iranian chronic HBV patients. This study intended to evaluate the prevalence of co-infection with HCV or HIV in chronic HBV patients who were referred to the Gastrointestinal and Liver Ward of the Taleghani Hospital, Tehran, Iran.

## 3. Patients and Methods

Two hundred and sixty four chronic hepatitis B patients who were patients in the Gastrointestinal and Liver Ward of the Taleghani Hospital between 2006 and 2010 were enrolled in this descriptive cross-sectional study. Written informed consent was obtained from all patients prior enrollment in this study. After obtaining each patient's demographic information and records of any risky behavior, including intravenous drug abuse, dangerous sexual contacts, cupping, hemodialysis, blood transfusion, tattooing, needle stick injury, dentistry operations, and use of shared razors, blood specimens were collected from each patient. Serum was separated from whole blood and tested for the level of liver enzymes, e.g., alanine transaminase (ALT) and aspartate transaminase (AST), by using an auto-analyzer (Liasys, Germany). The rest of the blood sample was stored at -70°C for further serologic tests. For confirmation of HBV infection, an enzyme-linked immunosorbent assay (ELISA) technique (Diapro, Italy) was used for detection of hepatitis B surface antigen (HBsAg) and anti-hepatitis B core protein antibody (anti-HBcAb). To determine the status of conversion from hepatitis B e antigen (HBeAg) to hepatitis B e antibody (HBeAb) in patients, we used an ELISA technique (Diapro) designed for HBeAg and anti-HBeAb. In the next phase of this study, the infection of these patients with HIV and HCV was assessed using an ELISA technique (Diapro) for the presence of anti-HCV and anti-HIV 1 and 2 antibodies. Samples with a positive result were tested again for confirmation. Data were analyzed by using SPSS 19 statistical software (SPSS Inc, Chicago, Illinois, USA).

## 4. Results

A total of 264 patients were enrolled in this study, including 184 men (70%) and 78 women (30%). The mean age of the patients was 41.58 ± 13.95 years (range: 11 to 77 years). All enrolled patients had prior knowledge of their infection, but for confirmation of their infection status, we tested them again for HBsAg and HBcAb. With regard to their status conversion from HBeAg to HBeAb, 32 patients (12%) were positive for HBeAg and negative for anti-HBeAb, 221 patients (84%) were anti-HBeAb-positive and HBeAg-negative, and 11 patients (4%) were negative for both markers(Table) HCV antibody was detected in 12 patients. In these patients, history of drug abuse (38.5%) and high-risk sexual contacts (33%) were the most-cited risky behaviors, and blood transfusion (4.3%) and hemodialysis (0%) were the least frequent high-risk behaviors. Only 1 patient was HIV antibody-positive. This patient was a 31-year-old man and an intravenous drug abuser. He also had a history of hemodialysis and periodontal procedures. He had no history of high-risk sexual contacts, use of shared blade razors, cupping, and tattooing. Interestingly, in the group of patients who had only HBV infection, the most prevalent risk factor was periodontal operation (28.8%), whereas intravenous drug abuse was really low in this group (3%) compared to HCV-co-infected patients.

**Table 1:** Patient Demographic and Serologic Information

	**HBV Alone**	**HBV+HCV**	**HIV**	**Total**
Gender				
Male	173	10	1	184
Female	76	2	0	78
HBeAg, No.^a^				
P	32	0	0	32
N	219	12	1	232
HBeAb, No.^a^				
P	208	12	1	221
N	43	0	0	43

^a^ Abbreviation: HBeAg, hepatitis B e antigen; HBeAb hepatitis B e antibody

## 5. Discussion

The results of this study showed that in chronic HBV patients, the prevalence of HCV is higher in comparison to HIV. Studies on HBV with HCV and HIV co-infections are increasing and have attracted the attention of researchers all over the world. HBV and HCV are among the main causes of liver problems. In addition, faster disease progression and higher occurrence of liver problems such as cirrhosis and hepatocellular carcinoma have been shown in co-infected patients compared to patients with only chronic HBV infection [7][12][13]. On other hand, the treatment regimen for patients with HCV co-infection in chronic HBV patients is different from that for patients with only HBV infection [9]. In addition, clinical studies have shown that HIV co-infection in chronic HBV patients can alter the natural course of the disease and treatment choices [14][15][16]. In the current study, a comparison of chronic HBV patients without co-infection and those with HCV co-infection showed that co-infected patients were more likely to be engaged in high-risk behaviors. Interestingly, while intravenous drug addiction was the most prevalent risk factor in co-infected patients, we observed that co-infected patients mentioned a history of sharing razors more frequently than patients infected only with HBV. In addition, the majority of patients reported periodontal procedures as a part of their medical history, which points to the possibility of transferring the infection through this route. A study of age distribution showed that the majority of non-co-infected patients were between 30 and 40 years of age, while co-infected patients were between 40 and 50 years of age. We could imply from this observation that the younger subjects, with higher health standards, could be less prone to co-infection.

In other parts of the world, researchers found different rates of co-infection. In a study performed in Kenya on HIV seropositive patients, the investigator reported that 23 patients (6%) had co-infection with HBV and HIV, 4 patients (1%) were co-infected with HCV and HIV, and 1 patient (0.25%) was infected with all 3 viruses [17]. In a study performed in the emergency department of a local hospital in Michigan, USA, investigators found that there was no co-infection and the prevalence of HBV, HCV, and HIV was 0.7%, 4%, and 0.8%, respectively [18]. In a study of drug abusers in Georgia, researchers reported that the infection rate for HBV, HCV, and HIV was 55.2%, 68.8%, and 1.7%, respectively [19]. In studies performed in Iran, researchers reached different rates of co-infection. In a study of hospitalized drug abusers in Kashan, Iran, Shariff and colleagues observed that 1% of inmates had a co-infection of HBV and HCV, but none of the inmates had triple co-infection [10]. Alavi and colleagues performed a study on co-infection of HBV and HCV with HIV in 104 HIV-positive drug addicts who were hospitalized in the infectious ward of the Razi Hospital, Ahvaz, Iran, between 2001 and 2003. They found that the prevalence of infection with HCV and HBV among these patients is very high (74.4% and 44.23%, respectively) [9]. In 2004, Torabi studied 162 hemophiliacs who lived in the eastern Azerbaijan province, Iran, for infection with HIV, HCV, and HBV and their prevalence was 0.6%, 51%, and 1.18%, respectively [11]. In light of the results reported in this study, we recommend that screening for HIV infection should be only executed for chronic HBV patients with a history of risky behavior, while screening for HCV co-infection should be mandatory for every chronic HBV patient.
